# Gender-Based Violence in Adolescent Dating from a Medical Perspective: A Qualitative Study of the Needs Felt in Primary Healthcare Centres

**DOI:** 10.3390/healthcare10010017

**Published:** 2021-12-23

**Authors:** Isabel Cuadrado-Gordillo, Guadalupe Martín-Mora Parra

**Affiliations:** Faculty of Education and Psychology, Department of Psychology and Antropology, University of Extremadura, 06071 Badajoz, Spain; guadammp@gmail.com

**Keywords:** gender-based violence, adolescence, primary healthcare, qualitative

## Abstract

Violence in adolescent dating has become a worrying public health problem. Research carried out on the issue has focused on identifying the causes of this phenomenon. However, difficulties have been found in designing and implementing effective prevention programs. In this context, primary healthcare physicians are one of the most important figures in screening for and detecting this phenomenon, since, in many cases, they are the first to have contact with the victims. The present study focuses on the qualitative analysis of a series of interviews carried out with 95 primary healthcare physicians in Extremadura, Spain. These interviews addressed various questions related to the theoretical and practical knowledge that the physicians have about the topic. The analysis of their responses reveals some of the strengths and weaknesses of the Spanish public health system, at the same time as pointing to what is needed to be able to improve comprehensive intervention for the victims, from the moment they arrive for their first consultation until they are treated and referred to different health specialists.

## 1. Introduction

Violence is a health problem that affects different kinds of interpersonal relationships and causes numerous consequences for the victims—physically, emotionally, and psychologically. One of the most prevalent forms of violence in today’s society is violence in couple relationships, and this phenomenon can be found all over the world, at all socioeconomic and cultural levels, and in any ethnic group [1,2]. In this sense, the prevalence of violence in adolescent dating relationships is high, even exceeding that of violence in adult couple relationships [3,4].

Concern over this phenomenon has led studies over the last decade to focus on the search for predictive and explanatory indicators [5,6,7]. These studies have been carried out from the perspective of both the aggressors [8,9,10,11] and the victims [12,13,14,15]. Nonetheless, despite all the research that has been conducted, violence in adolescent couples, far from decreasing, seems to continue to increase, with high rates of psychological violence as well as physical and sexual violence [16]. In this sense, although the prevention programs implemented seem to have had the effect of modifying adolescents’ attitudes towards gender-based violence as well as increasing their general knowledge about the phenomenon, there does not seem to be any reduction in their violent behaviour after participating in these programs [17].

In view of the above, as well as of the importance of the problem and its consequences for the victims, there is an evident need for a change of focus in the studies and analyses that are being carried out. To date, few research studies have focused on analysing one of the most important perspectives for the early detection of and intervention in this phenomenon—the point of view of the primary care physicians in healthcare centres. In this sense, the present study’s objective was to analyse the knowledge, strengths, weaknesses, and needs that the public health system and healthcare professionals have by giving the primary healthcare physicians themselves the role of protagonists.

Primary healthcare is an accessible service for any medical problem. It coordinates different general and specialised services and offers personalised and continuous care to all users throughout their lives [18]. The physicians in this service have a privileged position for both early detection and treatment. Among other reasons, this is due to their knowing the patient, their accessibility, and their ability to offer comprehensive care [19]. In this regard, some authors have reflected on how adolescents are more likely to attend a primary care centre rather than a specialised mental health centre to treat possible problems [20]. This may also be more often the case when they begin to show symptoms that they cannot explain, and which are, in most cases, unassociated with any victimisation situation. Indeed, various studies have revealed that adult female victims of gender-based violence go to medical services more, have a poorer general health status [21,22], present greater stress, and tend to use more antidepressant medications [21,23]. In the phenomenon of adolescent dating violence, therefore, both primary care and emergency services are in a privileged position to screen and detect problems, educate and guide the families, and refer the victims to such specialised services as mental health for intervention [24].

The potential negative consequences that violence and abusive behaviour have on adolescents mean that physicians need not only to be aware of this problem but to actively look for signs that may indicate this abuse, regardless of the age of the patient. Repeated complaints, or the absence of a clear clinical cause, can be signs of abuse, especially when these problems occur continuously over time [25]. Given the above, many medical organisations recommend the routine use of screening tools as part of primary care medical services [26]. Despite this, many victims go unnoticed. Indeed, only a small fraction of abused victims are detected in consultations, and in many cases this detection comes with a delay of up to 10 years from the beginning of the abuse [27], thus aggravating the consequences suffered by both the victim and their particular social environment. These situations can be even graver for adolescents, since the protocols used in primary healthcare services are focused on adult women victims, thereby leaving aside all the victims who do not fit that category. Likewise, it is possible that many physicians do not have the sufficient preparation, skills, and training to care for patients affected by the consequences of violence [28].

Taking into account the aforementioned limitations, the purpose of this study was to analyse a series of interviews with physicians of the primary healthcare services of the eight health areas in the Region of Extremadura (Spain) about adolescent couple violence. From this analysis, the objectives of the present study were:

i. To identify and integrate the perspectives that physicians in this field of healthcare have, specifically about victimisation in adolescent couples, addressing aspects related to their definition of and knowledge about abuse in this age range, how they identify the victims, the commonest symptoms, possible approaches to the problem, the level of their own involvement that they can accept, and the problems they encounter (both at a personal level and in the organisation and coordination of the services involved).
ii. To analyse the possible solutions to the problems found from the perspective of the primary public healthcare service physicians.

The questions asked during the interviews were divided into four categories that integrate all the aspects addressed in a comprehensive manner. The present work is, as far as we know, the first qualitative study to explore the components of victimisation in adolescent dating that has been carried out in Spain from the perspective of public health physicians.

## 2. Materials and Methods

### 2.1. Study Design

This study used interviews with questions specifically oriented towards the topic, together with questions of analysis and follow-up to ensure an adequate understanding of the physicians’ opinions. The physicians were asked a total of 14 questions. These questions were drafted after an analysis of the data collected in the first phase of the research, which had covered a total of more than 2600 adolescents of 14 to 18 years in age. The analysis of those data allowed an in-depth understanding to be formed of the problem of gender victimisation and its characteristics. Likewise, the extensive investigative work carried out made it possible to analyse the different information and support sources available to the primary health care centre professionals (physicians) to assist them in addressing perceived situations of violence. In this sense, it was found that the norms and protocols for comprehensive care in cases of gender violence were designed for the screening of adult couples but did not consider specific indicators of suspected gender abuse in adolescent couples. The researchers, with the basis of the existing protocols and information for adults, prepared a series of questions oriented to the information that they wanted to obtain. These questions were submitted to experts in primary care medicine for review. Finally, the 14 questions that made up the final interview were selected. Two examples of the general questions asked are: “How can abuse in adolescent couples be defined?” and “To what extent do you think alcohol and drug use can cause or enhance situations of violence in adolescent couples?”. Two examples of the specific questions are: “What level of involvement can a primary healthcare physician assume?” and “What indicators warn of the perpetration of situations of abuse in adolescent couples?”. An outline of the interview is given in Table 1.

With the prior authorisation of the physicians, all the interviews were recorded in an audio file, respecting the anonymity of the participants. These files were later transcribed into Word format and analysed in detail. In this analysis, a series of categories were created from the questions asked and the analysis of the responses given by the physicians. With this, the percentages of agreement were also obtained for each of the responses.

### 2.2. Participants

A total of 95 physicians of the primary healthcare services of the Extremadura public health system (SES) were interviewed. The primary healthcare centres were selected to cover all 8 health areas of the region of Extremadura (Spain). The participants were mostly experts with more than 20 years of service. In addition to the willingness shown by these professionals, without exception we found them to express great motivation and involvement in the study.

The 19 centres visited correspond to the populations of Extremadura with the greatest numbers of inhabitants. Of these, 10 belong to the province of Badajoz, and 9 to the province of Cáceres. Each was the main representative of one of the 8 health areas that structure the healthcare system in the region of Extremadura. With this, all the socioeconomic and cultural characteristics of the population in Extremadura were covered, in both rural and urban areas in each province. The participants were about equally divided between women and men.

### 2.3. Procedure

Prior to beginning the study, authorisation was requested from the management of the 8 health areas that had agreed to participate in the research study, and legal advice and permission were granted by the Health Care Bioethics Committee.

Once this authorisation had been obtained, the coordinators of each of the health centres were contacted to make an appointment presenting the objectives and purposes of the research study. Before starting the formal interviews, all the participants were informed about the objectives of the study, the content of the interviews to be carried out, and how long it would take to complete the interview, as well as guaranteeing their anonymity. Likewise, both the research objectives and the instruments used were analysed and approved by the Bioethics Committee of the University of Extremadura (Spain) (Ref. 18/2017).

The data collection process began in 2018 and continued throughout 2019. The researchers personally went to each of the health centres and Psychological Support Points (PAP) to conduct the interviews. The interviews were carried out in the health centres where the different medical professionals work. We ensured that the necessary time to carry out the interviews was available, with all of the centres agreeing that the interviews would be done at the end of the morning, once patient care was finished. The interviews were conducted in quiet environments without any interruption until the end of the interview. Each of the interviews lasted approximately 90 min and were conducted in Spanish, recorded in audio with the prior authorisation of all participants, and later transcribed for their analysis.

### 2.4. Analysis

A deductive-inductive thematic analysis of the transcripts was used in order to identify, analyse, and report patterns within the data. This analysis is based on the application of a total of six different phases that allow the content and meaning of the patterns found in the set of data analysed to be exposed. These phases include familiarisation with the reports, the generation of the first codes, the search for the underlying topics in the data, a review of the general topics found, their definition and naming, and, finally, constructing the report [29] (Braun and Clarke, 2006).

**Figure 1:**
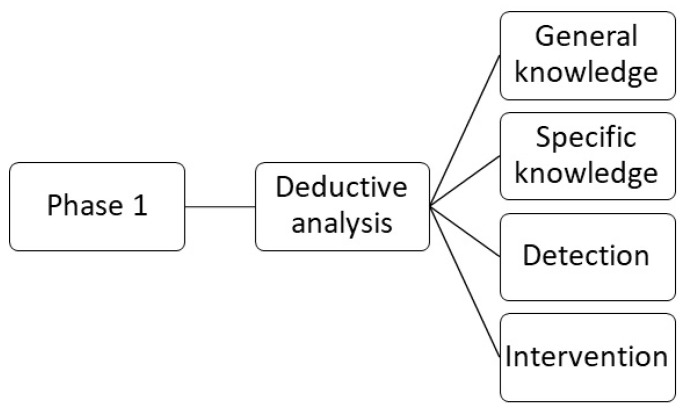
First phase of the analysis.

The first step has a deductive approach. After familiarisation with the data through various readings and re-readings of the transcripts, a preliminary code was developed in the first phase of analysis (Figure 1). Subsequently, the transcript was coded using four categories—general knowledge about adolescent dating violence, specific knowledge about adolescent dating violence, early detection of victimisation in consultation, and intervention.

The second step has an inductive approach. Specifically, the previously explained coding based on four categories was applied freely and without restrictions to create as many inductive codes as possible from the data. These codes had to be interpreted by the researchers, who then assigned them labels based on the information that the physicians expressed. Once the codes had been applied, the information was summarised in short sentences. For example, one participant comments, “I, well, I think there is no differentiation between adults and adolescents. The abuse is the same… in the end it is to control, the mobile phone or something else”; this was coded as “Definition of violence in adolescent dating”.

**Figure 2:**
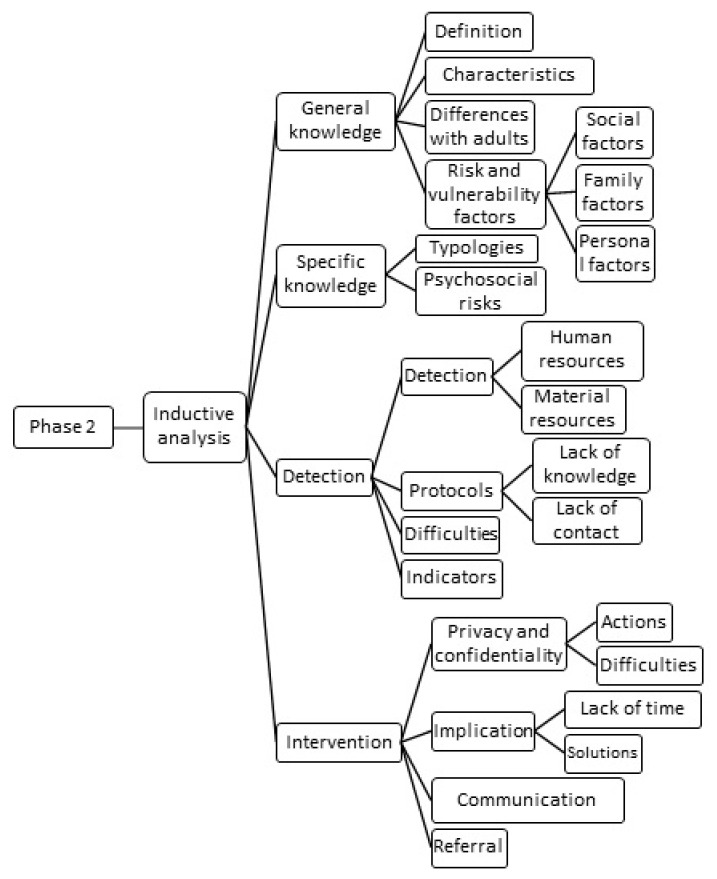
Second phase of the analysis.

Later, these codes were used to create topics and subtopics during the second analysis phase (Figure 2). This step again required interpretation on the part of the researchers in order to establish the relationships between the previously established discrete inductive codes. With this, the classification of the codes was carried out based on their actual content, as well as depending on the guide provided by the questions on which the interviews were based. For example, a participant explained, when answering the question of general knowledge about violence in adolescent dating relationships, “They, maybe it is an abuse of what they said before what we can see on Facebook, etc., To look at WhatsApp, but he loves me a lot and such…”; this answer was coded as “Characteristics” instead of “Definition”.

During the process of analysis, the initial code list was progressively redefined according to the inductive codes found. These codes were later organised into topics, which, additionally, were structured, in some cases, into subtopics following the initial code list.

The researchers analysed all the transcripts and established the initial code list, subsequent code redefinition, and categorisation into topics and subtopics. At a second moment, the preliminary work carried out was reviewed again to guarantee the validity and reliability in the coding and interpretation of the data, making various contributions and clarifications.

## 3. Results

This section is divided into subheadings. It should provide a concise and precise description of the experimental results and their interpretation, as well as the experimental conclusions that can be drawn.

Comments by the physicians about adolescent dating violence were used to identify various topics. For each category, the meaning of the category will first be explained, then the topics and subtopics included in each category will be described and some examples will be given. Finally, a summary table (Table 2) is presented, which includes the main topics analysed in the four categories constructed and the percentages of the responses that the physicians gave to each.

### 3.1. Category 1. General Knowledge about Adolescent Dating Violence

To identify violence in adolescent dating, it is first necessary for the physicians to have basic notions about the phenomenon and its implications. This general knowledge can be divided into (1) definitions, (2) characteristics, (3) differences from violence in adult couples, and (4) risk factors and vulnerability.

#### 3.1.1. Topic 1. Definition of Adolescent Dating Violence

Some of the physicians agree in defining the phenomenon as being similar to abuse between adult couples. Nonetheless, many of them agree that there are also some differences that must be taken into account. For example, one physician mentioned that the definition of violence in adolescent couples is the same as that given to adults, although it does have specific characteristics and implications that physicians must know in order to detect it:

“In principle, the definition of gender abuse is the same, then for adolescence it has different components… but the definition of gender abuse is the same.”

#### 3.1.2. Topic 2. Characteristics

These are traits that are associated with adolescent dating violence. Knowledge about these traits is essential for physicians since it will allow them to recognise this phenomenon when an adolescent comes for consultation reporting certain events that, on many occasions, the victims themselves do not associate as being forms of abuse. For the most part, the physicians interviewed manifested the importance that the aggressors’ control, social networks, and ICT, as well as pornography, have on the specific violence that takes place between adolescent couples. For example, one physician mentions:

“Social networks and such are selling us a type of relationship in the adolescent couple of being together all the time, but, above all, of having sexual relations and this is very much influenced by what boys and girls see in pornography. That aggressiveness, that humiliation. It seems to me, themselves, as this is a modeling thing right? So relationships are very polarised towards machismo, even women see as being normal. When she is with a person… let’s say, who does not follow her canons, he is a softie.”

#### 3.1.3. Topic 3. Differences from Violence in Adult Couples

The definition of the phenomenon implies knowing its specific characteristics when it occurs between adolescents. In this sense, most physicians indicate that this phenomenon is related to control, isolation from the rest of the group, and the use of social networks for abuse. For example, one indicates:

“They say: he loves me very much and that is why he controls my mobile phone.”

On the other hand, another physician at a different health area from the previous points out:

“In adolescents, mobile phones are used more, by taking away friends. A slightly more different control. Yes, indeed. That I think is more significant than the frequency.”

#### 3.1.4. Topic 4. Risk and Vulnerability Factors

These are factors that can be associated with and predict involvement in abusive relationships during adolescence. Although many of the physicians indicate that there is no specific profile that can be linked to abuse, they do find risk and vulnerability factors that can be used as a guide for its detection during a consultation. Among the risk factors most mentioned are (1) social factors, (2) family factors, and (3) personal factors.

Subtopic 1. Social factors. Most physicians indicate the important role played by social networks and the media. For example, one explains:

“I think that the main risk factor is in what you said before, in the media, social networks, the pattern of behaviour that they try to imitate because it is idealised. This malevolent program Hombres, Mujeres y Viceversa (Men, Women and Vice Versa). Malicious, because it talks about the relationships between men and women, and they insult each other, and they begin to…”

Subtopic 2. Family factors. Many physicians explain that, more than the economic or cultural level, the aspect that is truly associated with violence in adolescent couples is that of broken families and a lack of support. For example, one notes:

“More than the economic level, it is the destructuring. They can have a very high economic level and also be unstructured.”

Subtopic 3. Personal factors. Factors directly related to the adolescents. In this regard, a large portion of the physicians interviewed point out that the adolescent’s self-esteem, and factors such as the consumption of alcohol or drugs, may play a relevant role. However, many of them specify that substance use is more related to the aggravation of the problem, and therefore is not a precipitating factor. For example, one explains:

“I think it does not precipitate. That it may be another factor that aggravates, yes, but as the main cause I think not. Not as the main cause.”

### 3.2. Category 2. Specific Knowledge about Adolescent Dating Violence

To be more precise in recognising the phenomenon of adolescent dating violence, the physicians indicate that, beyond having some general knowledge, it is necessary to have specific knowledge such as (1) the types of abuse and (2) the psychosocial traits of the victims.

#### 3.2.1. Topic 1. The Commonest Types of Abuse between Adolescents

The physicians generally note that it is important to take into account that adolescents do not live with their partners, since they are mostly minors, and that this implies a lower risk of physical violence and a greater presence of emotional abuse and sexual blackmail. For example, one explains:

“I think that the definition you gave before is very good. In this case, I consider that… that for, in, in adolescents perhaps it is rather, the verbal, verbal and psychological violence rather than the physical one. Physical violence is stronger among adults. Sometimes it also exists in adolescents, but then the girl can also respond to the boy, but I think that is when the abuse is more advanced. I would define it rather as psychological and verbal violence.”

#### 3.2.2. Topic 2. Psychosocial Traits of the Victims

In this regard, most of the physicians point to the same factors that they had previously indicated when answering the question about vulnerability factors. That is, they usually notice that the victims tend to come from broken families, this disruption being independent of economic factors or social class. In this sense, the presence of violence in any social environment is highlighted, with family support, education, and the transmission of appropriate values becoming more important. For example, in this regard, one physician indicates:

“What I see is the family atmosphere. That is noticeable. If the mother has an old-fashioned family relationship, father, mother where the father is the one who is there to work, the mother is there to take care of the children, such as is seen a lot. Well, they see it as their logic of life. If you see it every day at home, you will follow it.”

### 3.3. Category 3. Detection of the Phenomenon of Adolescent Dating Violence in Primary Healthcare Centres

Once the importance of having general and specific knowledge about adolescent dating violence has been established, the physicians focus on detecting the phenomenon by explaining what resources they can use as healthcare physicians. In this sense, essentially, there are four different topics they mention: (1) reliable sources of information; (2) protocols for observing behaviour, signs, or symptoms; (3) difficulty in detecting the problem; and (4) warning indicators.

#### 3.3.1. Topic 1. Information Sources

These are places or people to go to when there is suspicion of violence in adolescents. Many of the physicians point out the importance of having reliable sources of information to turn to for help and guidance, even more so considering that they are not experts in physical and psychological indicators of abuse. In this sense, the accessibility to (1) human resources and (2) material resources are indicated as being sources of information.

Subtopic 1. Human resources. All the physicians concur in pointing to their colleagues from the area of mental health—psychologists and professional social workers who are more familiar with the subject. Likewise, many physicians indicate that it would be helpful to have experts (psychologists) within the primary healthcare area that are able to respond quickly. Relying on psychologists who are physically and organisationally located elsewhere greatly hinders consultation in the field of health, and consequently the early detection of victimisation. For example, one notes that:

“There have been times when we have used the Casa de la Mujer (Women’s House) psychologist because what is clear is that this has to be a specialised treatment. The person who is in the mental health unit is of no use, they are of no use to us, it has to be a person with specific training. So, we have used the Casa de la Mujer one. There have been times that the psychologist was only there for the women who were admitted, well admitted, who were there living together, and she, well, to do us a favour, assisted us.”

Subtopic 2. Material resources. Material aid to turn to when there is suspicion of violence. The importance of having a good and detailed medical history is noted so that any healthcare provider (including not only physicians but also nurses and other professionals of the area) who attends the person when they come for consultation can refer to the previous health history of that patient. On many occasions, it is found that the victims may be people who are hyper-users of health services who show no specific physical symptoms, and this may be reflected in said medical history. For example, one physician notes:

“Sure, the history written down. The history that we have written down in the medical history. A hyper frequenter, there, there is usually something behind it. But a girl who comes today, because today she has a pain in the wrist and such, I don’t ask her if she has…”

#### 3.3.2. Topic 2. Action Protocols

These are protocolised guides that indicate the steps to follow to detect and intervene in cases of adolescent dating violence in primary healthcare. Most physicians indicate that they do not currently have specialised protocols for adolescent couples. In this sense, most of the standardised protocols to which they have access are aimed at adult women. For example, one notes:

“I have not had any case, or I have not detected one, thus, in consultation, but I also do not know what I should do because if the adult protocol… it is that it is a very, very… issue, because it is a minor, because… The protocol, must be different from that of the adult woman, right?”

#### 3.3.3. Topic 3. Difficulties in Detecting the Problem

These are factors associated with the inconvenience physicians have in suspecting and detecting cases of violence in adolescents during consultations. Two subtopics arise in this regard: (1) a lack of knowledge and (2) a lack of contact.

Subtopic 1. Lack of knowledge. A large portion of the primary healthcare physicians from the public health system indicate that, despite not having the necessary knowledge in many cases, if they could have more time to carry out an adequate evaluation, the problem could be detected. Nonetheless, in most cases, the high number of patients who have to be seen each day makes this impossible. For example, in this regard, one notes:

“I don’t think I have the necessary knowledge, but I think that, if you have done a good exploration, with some good complementary studies, and everything turns out negative, there is a problem. That’s when you think about it… When everything is normal at an organic level, tell me… If you stop, if you have time, yes. Hey? If you stop, yes. But it’s difficult, we don’t have the time or the knowledge.”

Subtopic 2. Lack of contact. Many physicians in Extremadura point out that another problem that arises for the detection is the lack of contact they have with the adolescents. As they note, when children go from pædiatric to primary healthcare services, they usually lose these patients. Few adolescents regularly visit their doctor, and this consequently complicates the detection of these types of problems. Likewise, when they do go to the physician, care is complicated because of the limited time dedicated to each of the patients, a fact that prevents any detailed investigation from being carried out. In this sense, some physicians note, as a possible alternative, the relevance of creating a specific service for adolescents (similar to the one in certain countries such as Colombia) where they can go alone, without family members, thereby fostering trust. Thus, a physician points out:

“In Colombia… I think there are many formulas, many, many. Let’s see, there are formulas right? In Colombia, for example, in primary healthcare centres there are thus two, three physicians, or female physicians, who are left for consultations only with adolescents. Dedicated only to adolescents.”

#### 3.3.4. Topic 4. Alert Indicators

These are signs that indicate the presence of a problem in adolescent patients and that may be related to adolescent dating violence. In this sense, all the physicians seemed to agree that the most relevant symptoms which usually indicate some type of abuse are somatisation (headaches, pelvic pain, anxiety, palpitations,…). For example, one physician, when talking about this topic, indicates:

“I however consider headaches. Headaches, pelvic pain, there is always pelvic discomfort, that they have pelvic discomfort. What do I have? Well maybe I have a urine infection or something, or maybe I have vaginitis. Well, they don’t know the term, but more or less they always, tell you that it is more in the pelvic area, and headaches in my experience.”

### 3.4. Category 4. Intervention in the Phenomenon of Adolescent Dating Violence in Primary Healthcare

Finally, the physicians indicate some of the most important aspects to consider related to the intervention that they, as primary healthcare physicians, can carry out after detecting a case of violence in adolescent couples. In this sense, they indicate, as being determinants: (1) intimacy and confidentiality, (2) involvement, (3) communication, and (4) referral.

#### 3.4.1. Topic 1. Intimacy and Confidentiality

This is the climate of trust that is created between the physician and patient that allows the latter to speak with their physician freely, without fear of censorship or moral judgement. Here, two subtopics arise: (1) actions to be taken to achieve confidentiality and intimacy, and (2) difficulties in achieving confidentiality and intimacy.

Subtopic 1. Actions to achieve confidentiality and intimacy. Behaviour and gestures that physicians can perform during the consultation to get the adolescents to trust them and tell them what is happening or how they feel. Most of the physicians indicate that it is essential to create an intimate space in which they can be alone with the adolescents, without the people who usually accompany them (mothers and fathers), so that they feel that what they are saying is confidential. For example, one explains:

“I take them outside, the family. I ask the child, do you want them to be there, do you want them to be outside and I take them out, even if they are not 16 years old, eh? At 14, do you care to go out? … It’s the only way.”

Subtopic 2. Difficulties in achieving intimacy and confidentiality. This section refers to the barriers that primary healthcare physicians encounter against achieving, during consultations, a climate of trust and confidentiality that encourages adolescent patients to tell their physicians about their problems. Although most families usually collaborate with the physicians, some physicians indicate that since they are dealing with minors, they do not feel prepared, or that it is impossible to create a doctor–patient space due to legal restrictions. For this reason, they try to overcome these difficulties by implementing some resources that help them to gain the trust of the parents at the same time as they achieve that of the minor. Nonetheless, it is not always possible to achieve complete confidentiality. For example, one notes:

“There has to be, has to be an adult. There are times when, when they want to tell you something strange, you ask the mother’s permission, who is the one that usually accompanies in the case of a girl, or the father in the case of a boy. This is usually the case. So if they want to tell you something, you ask the father or mother to go out, but then the female nurse or male nurse has to come in with you. We do not see them alone. In other words, there is no one-to-one confidentiality.”

#### 3.4.2. Topic 2. Involvement

This is the level of involvement that a primary healthcare physician can assume in the detection and intervention in cases of adolescent dating violence. In this topic, there are two subtopics: (1) time constraints and (2) limitations to the solutions.

Subtopic 1. Time constraints. Most of the physicians interviewed explain that their involvement with the issue is maximum, but they point out that they usually do not have the time or the necessary preparation to be able to materialise said involvement. These time problems are related to both the number of patients they have and the amount of time that they can officially dedicate to each of the consultations (approximately 6 min). For example, some note, in this regard:

“What… a 5 min involvement. Five minutes is little time.”

Subtopic 2. Limitations to the solutions. Some physicians point out that, with goodwill on their part, they can try to implement some solutions to the problem of time. However, these solutions have their own difficulties that end up, far from resolving the problem, causing it to continue, and in many cases resulting in the loss of the patient. For example, in this regard, one explains:

“I may not have time today, but I can make an appointment for you tomorrow, or the day after tomorrow at 1.30 pm. And if you are interested, you come, and if you have no interest, you do not come. And when I have given you an appointment, if you don’t come, I have lost you. But I know that there are people who want immediacy. So when you see me now, and I need half an hour, and I don’t have half an hour. I don’t have half an hour. I don’t even have 10 min for that patient. I don’t have it.”

#### 3.4.3. Topic 3. Communication

This refers to the form of approaching communication with the families of the adolescents. Most of the physicians indicate that, since these patients are minors, once a case of dating violence has been detected or suspected, it is necessary to speak with the families. Parents should be aware of the situation, as they need to approve the referral to specialised services to continue with the intervention process. In this sense, most physicians indicate that, in general, communication with families is easy. In talking about these aspects, one says:

“But they accept that if you send her to the mental health unit, that’s fine with them. When it has come to this it is because they have not been able to deal with it at home. Any door you open for them…”

#### 3.4.4. Topic 4. Referrals

This is the process of referral to the specialists responsible for interventions in cases of gender-based violence detected in adolescent dating. In this regard, all the physicians agree that the only resource they have is the mental healthcare service. Likewise, all of them agree in indicating that this service is usually so saturated that it cannot provide the immediacy and speed that these cases need. With this, they explain that, in many cases, the only solution is to have a private, professional psychologist that not all patients can afford. For example, some comment:

“Here we only have mental healthcare and the relationship with the psychiatry department” … “The waiting list…, a referral…, yes, there is the possibility of referral, but honestly it is not much use either. A two-month waiting list for an interview… that’s worth nothing” … “A private psychologist, that’s the solution we found, but, of course, not everyone can access it.”

## 4. Discussion

This study has explored the point of view of primary healthcare physicians from health centres of the public health system of Extremadura (Spain) about the phenomenon of gender-based violence in adolescent dating. A series of semi-structured interviews were carried out that were later transcribed into Word format. The transcripts were then coded by following a method of deductive-inductive thematic analysis. The study contributes to understanding the strengths, weaknesses, and needs that exist within the field of primary healthcare with regard to knowledge about the characteristics, detection, and treatment of victimised adolescents in dating relationships.

Starting with the general knowledge that the physicians have regarding the phenomenon of adolescent dating violence, the interviews show that they are aware of the phenomenon, not only by demonstrating knowledge about its definition but also by explaining what its characteristics and differences are with regard to gender-based violence in adult couples. In this respect, the importance that physicians attach to social networks, the internet, and the media in general is especially relevant. Thus, many point out how abuse in adolescent couples is carried out to a greater extent through their mobile phones—monitoring social networks and making multiple calls or text messages. These physicians seem to coincide with an increasing number of researchers who point to the problem of dating violence committed through the internet and social networks [30,31,32,33]. In the same line, Ref. [34] notes how ICTs have redefined the limits of romantic relationships since they provide a broad terrain for conflict and abuse, especially relevant being the opportunities given to the aggressors to control their partners because of the absence of spatial limits [35] and the instantaneity of social media, being able to act from a distance and at any time of the day or night [36].

Nonetheless, the use of social networks is not the only fact of interest indicated by primary healthcare physicians. They also highlight the role that the media have in transmitting values that end up fostering violence. The ideals of romantic love that music and television transmit [37] are acquired and integrated by adolescents as being their own, thereby promoting both aggression and victimisation, as well as connecting with sexist stereotypes that are highly present in such social networks as Instagram or Snapchat [38]. These social networks (which are based on the use of images and photographs) favour the manipulation of the image itself to adapt to the prevailing fashion, and this is often idealised, unreal, sexualised, and adapted to unattainable social ideals [39].

With regard to their specific knowledge about this process, the physicians again show this to be well-adjusted and accurate, coinciding with the research that has been carried out on the phenomenon of violence in adolescent dating today. Thus, in their opinions, and taking into account their experiences in their consultations, the physicians note that the abuse occurs independently of the socioeconomic and cultural status of the victims and that this behaviour is more closely related to such aspects as a destructured family. In particular, they highlight the existence of unstructured families at low, medium, and high socioeconomic levels. This fact relates to other research findings that show that witnessing or suffering violence during childhood is linked to victimisation and aggression during adolescence and adulthood [12,15].

Despite the observations they describe, it is surprising that most of the physicians indicate that they do not feel prepared to carry out the tasks of detection, screening, and intervention with the victimised adolescents who come to their consultation. Although it is true that detecting a victim of gender-based violence is no easy task, especially given the lack of training that the physicians reveal they have, at the same time it was found that these health professionals themselves, in their responses, declare precise data that could serve to guide them in a fairly straightforward way to the detection of victims. For example, a large percentage of the physicians interviewed indicate that an adolescent who is hyper-frequent in their medical consultation and who does not present any physical syndrome—but does have somatic symptoms—is, in itself, a cause for alarm. Therefore, this supposed lack of knowledge, which points in the same direction as previous research [40], could denote a feeling of insecurity that goes beyond the lack of knowledge about the phenomenon itself (which, in fact, they seem quite familiar with). Thus, authors such as [41] indicate that, in Spain, the attention given by primary healthcare teams to victims of violence gives great importance to the involvement of these professionals in community initiatives. However, this involvement and commitment are totally voluntary and individual, which makes such initiatives hard to sustain. In this sense, the promotion of community activities that would involve all primary healthcare professionals could foster the feeling of self-efficacy in helping the victims [42]. Similarly, training courses based on scientific evidence published by experts [43,44,45,46] would not only help physicians acquire the skills needed to aid the victims but also promote their feelings of self-efficacy [47], and with this, increase their confidence.

Another variable to consider in analysing the feelings of insecurity that these physicians show in the detection of the phenomenon of adolescent dating violence is the lack of time to carry out extensive consultations that would allow for an adequate interview and exploration. In this regard, the physicians repeatedly report having large patient quotas, so that they can only dedicate a maximum of 6 min to each of them. This fact seems to make the creation of an adequate atmosphere of intimacy and confidentiality, which could help the victims share their concerns and problems, almost impossible. In this sense, it has been pointed out how the organisation of primary healthcare services in Spain encourages the establishment of doctor–patient relationships of trust, due to the possibility of seeing the same patients over long periods of time, and thereby, to a certain extent compensating the short time duration of the consultations themselves [48]. Nonetheless, this factor might be different in the case of adolescents. This age group does not usually have serious health problems, so they do not usually visit their physician regularly, which makes it impossible to foster that relationship of trust over time in the same way as is the case of adult patients. In this regard, the fact should also be noted that adolescents in Spain are switched from pædiatric to adult services at the age of 14. Hence, in many cases, they would hardly know their primary care physician.

The issue of confidentiality becomes even more complicated when the physicians explain the special situation of the adolescents who come for consultation. These individuals, as minors, usually come for consultations accompanied by their parents or caregivers, who tend to remain within the consultation while the adolescent reports their symptoms and problems. This fact constitutes another barrier to establishing an adequate doctor–patient relationship that encourages intimacy and confidentiality, even more so taking into account the circumstances surrounding victimisation in a dating relationship. In many cases, feelings such as shame or guilt can make adolescents not want to share certain, more intimate aspects of the couple’s relationship with their physician in front of their parents, and thus they omit relevant information or minimise the abuse suffered. Indeed, it has been pointed out that a large percentage of adolescents continue to show concern regarding the issue of confidentiality. Thus, many young people go so far as to not to seek medical help when they feel that confidentiality may be a problem, a fact that can be quite common considering that half of adolescent girls and boys have never discussed the issue of confidentiality with their doctor [49].

Relying on the families’ goodwill to provide a personal doctor–patient space in the case of underage adolescents can make it difficult not only to create an adequate atmosphere of trust but also to talk about confidentiality, which makes it hard for the physician to have the possibility of explaining, in detail, what such confidentiality is and how it is going to be involved in what is spoken about during the consultation. Likewise, even if this doctor–patient space is achieved with the adolescent, scientific evidence shows that many parents want to be subsequently informed of what was talked about during the consultation, seeing this fact as a parental right [50,51,52].

In contrast with the above, the analysis of the interviews in this study shows that primary healthcare physicians usually have the collaboration of the parents when the latter are asked to respect the doctor–patient space during a consultation with their adolescent children, with the exception of families from certain cultures or social strata. This fact differs from previous research that has noted major problems when healthcare physicians try to be alone with the adolescent patient [52,53,54]. An explanation for this difference could lie in the characteristics of the Spanish healthcare system, which allows the physician to get to know the families (first, the parents, and later the adolescent children). This makes it likely that the parents have already created a relationship of trust with the physician, meaning that they are more willing to leave their children alone with the physician.

Finally, another relevant finding of the present study is related to the need that the physicians express of having reliable sources of information to turn to when they meet an adolescent who fulfils the characteristics of the role of victim. In this sense, the physicians indicate needs related to human and material structures, i.e., to have psychologists who are readily accessible, as well as specific protocols designed for gender-based violence in adolescent dating.

The detection and screening of victims, and any subsequent interventions, seem to be greatly limited due to the restricted possibility of consultations that physicians can have with their colleagues because of the waiting lists and the fact that mental health services are not as accessible as primary care services. There seems to be no type of structure that coordinates the relationship between the different professionals involved in the proper care of victims, making the collaboration of these physicians purely voluntary, which points to yet another barrier. In this regard, some studies, such as that carried out by [55], suggest that, in cases of violence, it is necessary to create multi-disciplinary and multi-professional working groups that include professionals of all profiles who come into contact with the victims (nurses, primary care physicians, trauma physicians, social workers, surgeons, gynæcologists, etc.).

However, the formation of work teams dedicated to the detection of cases of adolescent dating violence may not be enough. The point made by the physicians that were interviewed about the lack of specific protocols aimed at adolescent victims reflects a lack of coordination in primary healthcare services. Without the existence of a figure or structure that coordinates, connects, supports, and distributes the work, as well as establishing clear objectives [48], it is difficult for work teams to be truly effective. Hence, one can state that any action of this type must be supported by the administration, also taking into account the needs and the barriers indicated by the primary healthcare service physicians, as well as the patients who are the principal objective to whom the intervention will be directed.

## 5. Conclusions

This study has highlighted the theoretical and practical knowledge that primary healthcare physicians have about the phenomenon of gender-based violence in adolescent dating, revealing this knowledge to be an essential part of the detection, referral, and treatment of the victims. With this, these findings have made it possible to attain the objectives set out for the research. This is both in the case of the first objective, the identification and integration of the medical perspective on violence in adolescent dating and its characteristics, and the second, the analysis of the problems found in primary care services. Furthermore, this analysis gives an idea as to what solutions might be implemented to strengthen the primary public health system in order to detect and intervene in cases of adolescent victims.

Specifically, the study establishes first- and second-order predictive indicators of vulnerability for the detection of situations of violence and victimisation in adolescent couples. It also identifies factors that may end up being converted into precursors of aggressive behaviours and indicators of suffering or carrying out actions of gender-based abuse. All of this allows the early screening protocols for aggressive behaviour patterns to be optimised in primary care at health centres in Extremadura (Spain).

The interviews analysed show that the physicians, far from being unaware of the problem, have data and information that could be of great help in the detection and treatment of the victims. One of the most relevant findings of this research is that the needs expressed by the primary healthcare physicians are related more to the provision of human and material resources specifically developed for this purpose than to the acquisition of general knowledge about the topic. Likewise, it is essential to have the support of the administration through the creation of coordination services that can connect all the parties involved, increase the physicians’ security and confidence in their own abilities, and strengthen the positive points that primary healthcare systems such as that of Spain already have. It is also essential to design specific protocols to help detect adolescent dating violence. Likewise, the time factor is very relevant in the initial diagnosis of victimisation, since the adolescents’ somatisation, which is the object of their consultation, can be very diverse. For this reason, the physicians repeatedly pointed out that the time they have makes it unfeasible to carry out any in-depth exploration of the different cases to determine the care that is needed. Finally, an agency that coordinates all the services involved and the actions to be taken could be a step forward for early detection and intervention in cases of abuse in adolescent dating.

## 6. Limitations

The present study may have possible methodological limitations, such as the self-reported nature of the data. Firstly, the participation of the primary healthcare physicians of the public health system was voluntary. It is, therefore, possible that they were more committed to the phenomenon of gender-based violence in adolescent dating, and therefore have greater interest and knowledge about the subject. Neither was it possible to control for the gender of the medical professionals, the men and women who attended the interviews, so that we do not know whether the said variable in the medical profession is a differential factor in relation to the commitment and concern that this phenomenon arouses in physicians. Moreover, having the opinions of psychologists with expertise in attending to the victims of violence, as part of the intervention following their detection, would have complemented the conclusions reached in the present study. These limitations could serve as a guide for future research aimed at expanding the knowledge about the subject.

## Figures and Tables

**Table 1:** Outline of the interview.

Category	Question
General knowledge questions	
Question 1	How could you define dating violence among adolescents?
Question 2	What are the risk and vulnerability factors of those who are subjected to this kind of violence?
Question 3	To what extent do you think alcohol and drugs could cause or enhance situations of violence in adolescent couples?
Question 4	What are the most common sources of information you consult to detect abuse in adolescent couples?
Question 5	What are the psychosocial traits which characterise dating violence victims that you see in your office?
Question 6	Do you use any protocol when you observe suspicious behaviour, symptoms, or signs of violence?
Specific knowledge questions	
Question 1	How do you work with minors on the topic of confidentiality between doctor–patient?
Question 2	How do you create a climate of trust with minors so they can tell you what they are suffering?
Question 3	What level of involvement can a primary care physician assume in these situations?
Question 4	How is the process of communication and understanding with the families?
Question 5	Thinking about the cases you have had in your medical office, what are the most common types of victimisation?
Question 6	Could we say that it is easy to be suspicious about the possibility of abuse in adolescents who come for a consultation?
Question 7	What indicators warn of the perpetration of situations of abuse in adolescent couples?
Question 8	Are adolescent victims referred to a specialised service?

**Table 2:** Summary of the percentage of the type of responses provided by the physicians.

Summary of Topics	Responses Provided by the Physicians			
Definition-Characteristics	Control aggressors–victims	Verbal and emotional violence	Same as adults	Others
	52%	13%	13%	22%
Characteristics	Destructured families	Media	Social networks	Pornography
	35%	22%	22%	21%
Risk factors	Destructured families	Social networks	Self-esteem	Others
	35%	40%	20%	5%
Typologies	Psychological	Verbal and emotional violence	Blackmail and control	Others
	61%	23%	12%	4%
Detection	Social services	Mental health	Lack of resources	Others
	35%	19%	8%	38%
Protocol use	Does not exist	Intuition		
	97%	3%		
Ease of detection	Easy	Yes, with time	Yes, intuitive	Others
	70%	24%	4%	2%
Indicator	Somatisation	Hyper-frequentation	Anxiety	Others
	50%	17%	11%	22%
Privacy and confidentiality	Lack of time	Problems with creating a physician–patient space	Necessity of information	Others
	44%	31%	11%	14%
Implication	Lack of time	Duty	High	Others
	49%	27%	19%	5%
Communication	Family collaboration	Depends on the situation	Complicated	
	87%	8%	5%	
Referral	Mental health	Lack of resources	Psychologists	Others
	64%	16%	14%	6%

## Data Availability

Not applicable.
